# Improved Base Belief Function-Based Conflict Data Fusion Approach Considering Belief Entropy in the Evidence Theory

**DOI:** 10.3390/e22080801

**Published:** 2020-07-22

**Authors:** Shuang Ni, Yan Lei, Yongchuan Tang

**Affiliations:** School of Big Data and Software Engineering, Chongqing University, Chongqing 401331, China; 20171674@cqu.edu.cn (S.N.); yanlei@cqu.edu.cn (Y.L.)

**Keywords:** Dempster-Shafer theory, coflict data fusion, improved base belief function, information volume, belief entropy

## Abstract

Due to the nature of the Dempster combination rule, it may produce results contrary to intuition. Therefore, an improved method for conflict evidence fusion is proposed. In this paper, the belief entropy in D–S theory is used to measure the uncertainty in each evidence. First, the initial belief degree is constructed by using an improved base belief function. Then, the information volume of each evidence group is obtained through calculating the belief entropy which can modify the belief degree to get the final evidence that is more reasonable. Using the Dempster combination rule can get the final result after evidence modification, which is helpful to solve the conflict data fusion problems. The rationality and validity of the proposed method are verified by numerical examples and applications of the proposed method in a classification data set.

## 1. Introduction

Dempster-Shafer theory (D–S theory) [1,2] plays a vital role for addressing uncertainty in medical diagnosis [3], target recognition [4,5], fault diagnosis [6], classification [7,8,9], clustering [10,11,12], risk analysis [13] and many other fields [14]. D–S theory can clearly measure the uncertainty of events, and then provide the basis for decision-making by the data fusion results. However, due to the complexity of data, evidence conflicts are often encountered in the actual data processing. In [15], the concepts of conflict from different perspectives are proposed to clarify what conflict is and from where the conflicts come. In [16,17], Zadeh points out that if a conflict exists between the subjects of evidence, classical evidence theory will often get the opposite results in its normalization process. Due to the nature of Dempster combination rule, it may produce results contrary to intuition [16,17]. Smets analyzes the ’jungle’ of combination rules and the nature of the combinations [18]. Classical evidence theory can not deal with conflict data effectively, which greatly restricts the promotion and application of evidence theory. Therefore, this paper studies the conflict data fusion.

Because the several pieces of evidence from multiple information elements are often inconsistent, the data is often in conflict. Many experts and scholars have done a lot of research on conflict data fusion. At present, there are many methods to solve conflict data fusion [19,20]. Part of the research focus on proposing new combination rules like inconsistent measure-based rule [21], combination rule considering evidence dependence [22] or improving the original combination rules using belief entropy-based method [23], fuzzy element [24], so as to improve the results of conflict data fusion. In [25], some basic principles are proposed after a systematic review of existed fusion rules, which can reasonably solve the fusion when there exits incomplete information. In [26], a new combination rule is proposed, which is based on the analysis and illustration of similarity collision. And this method aims to solve the problem of conflict. In [27], Decision making trial and evaluation laboratory (DEMATEL) method is proposed to merge conflicting data. New combination rule can effectively solve problems in recognition field. In [28], the improved combination rule of D-number is applied to emitter identification. In [29], a method is proposed to select the source behavior, which is based on a very general and expressive fusion scheme. The important advantage of this method is that it can clearly explain the assumption of the source. Furthermore, incomplete information should also be considered in conflict data fusion [30,31,32].

In addition, another method of conflict fusion is to manage the uncertainty in the evidence sources before evidence fusion. Entropy is a typical method for uncertainty measure and management [33]. In evidence theory, the belief entropy [34,35,36] or uncertainty measure of mass function [37,38] is used to address the information volume of evidence, so as to modify evidence sources. In [39], Deng entropy is proposed, which can not only deal with the uncertainty of basic probability distribution effectively but also correctly. Deng entropy, as a belief entropy, has been widely used in many applications such as risk analysis [40]. In [41], a Multiple-Criteria Decision-Making method is proposed, which is based on D Numbers and Belief Entropy. This method can deal with the conflict problems effectively. In [42], a novel belief entropy is proposed to measure uncertainty of basic probability assignments, which is based on belief function and plausibility function. This method can deal with the conflicts reasonably during information fusion. In [43], an improved method is proposed to combine conflicting evidence, which is based on the similarity measure (which can evaluate the similarity between two things) and belief function entropy.

Besides, constructing initial belief on each evidence can reduce the conflict between BPAs. In [44], base belief function is proposed, which can modify the BPAs to deal with conflict data fusion. Based on this, in [45], an improved method is proposed to manage conflict data by assigning an elementary belief. On the basis of the conflict data fusion strategy, the improved base belief function [45] and belief entropy [39] are used to solve the problem of conflict data fusion. The procedure of the proposed method is as follows. Firstly, the BPAs are modified by the improved method of base belief function. Secondly, belief entropy is used to calculate the information volume and to get the weight of each evidence group. Thirdly, the weight is used to modify the BPAs again. At last, the Dempster combination rule is used for data fusion.

The proposed method can solve the data conflict problems effectively which can get better combination result. The proposed method considers both the focus elements in the current evidence and the proposition in the power set space. In addition, the proposed method reallocates the BPAs for conflicting data, which also can solve some the initial BPAs that are zero value in each evidence group. At the same time, due to different information sources having different influence on the final results, the proposed method can distribute the weight according to the information volume of information sources. The final BPAs obtained by using the belief entropy can make the data fusion results more logical.

The following parts of this paper are organized as follows. In Section 2, we review some basic concepts. Then in Section 3, we propose an improved approach using information volume to weight basic probability assignment, so as to obtain a reasonable combination result using D–S theory. In addition, a few examples are given to verify the correctness of the proposed method. In Section 4, the classification experiments are presented to show the effectiveness of the proposed method. The open issues are given in Section 5. Finally, conclusions of proposed method are given in Section 6.

## 2. Preliminaries

In this section, some preliminaries are introduced.

### 2.1. Dempster–Shafer Evidence Theory

Dempster–Shafer theory [1,2], which is known as belief function theory, is the extension of the Bayesian subjective probability theory. The evidence theory was developed by Shafer, the concept of belief function is also introduced by him. Shafer formed a set of mathematical methods of “evidence” and “combination” to settle the uncertain reasoning. The D–S evidence theory does not need to know the prior probability, which can represent “uncertainty” well. In addition, D–S theory is widely used to deal with uncertain data. It is mainly applicable to information fusion, expert system, information and legal case analysis, multi-attribute decision-making analysis as an uncertain reasoning method. Its biggest characteristic is to use “interval estimation” instead of “point estimation” for the description of uncertainty information, so as to distinguish the unknown and uncertain aspects, accurately reflect the evidence collection, which shows great flexibility.

Let *U* be the frame of discernment (FOD). Basic Probability Assignment (BPA) is a mass function m which is $2^{U}\to \left[0,1\right]$ and satisfies

(1) $$m\left(\emptyset \right)=0,\sum_{A\subseteq U}m\left(A\right)=1$$

In FOD, belief function is defined as,

(2) $$\begin{array}{c} Bel\left(A\right)=\sum_{B\subseteq A}m\left(B\right) \end{array}$$

The plausibility function [46] is defined as,

(3) $$\begin{array}{c} Pl\left(A\right)=\sum_{B\cap A\neq \emptyset }m\left(B\right) \end{array}$$

The Dempster combination rule is a key step to combine the output of multiple principals. For two mass functions $m_{1}$ and $m_{2}$, the Dempster combination rule can be defined as follows:

(4) $$\begin{array}{c} m_{1,2}\left(A\right)=m_{1}\left(B\right)⊕m_{2}\left(C\right)\\ =\frac{\sum_{B,C\in \Omega ,B\cap C=A}m_{1}\left(B\right)\times m_{2}\left(C\right)}{1-\sum_{B\cap C=\emptyset }m_{1}\left(B\right)\times m_{2}\left(C\right)}, \end{array}$$

where a coefficient *K* is defined as follows:

(5) $$K=\sum_{B\cap C=\emptyset }m_{1}\left(B\right)\times m_{2}\left(C\right).$$

The advantages of the Dempster combination rule are mainly reflected in the case of less evidence conflict. However, the Dempster combination rule also has some disadvantages. If there is a high conflict between two pieces of evidence, the following defects will appear when using them: It may assign 100% belief to a small possible proposition, which will produce results contrary to intuition. It is also very sensitive to the allocation of basic reliability.

In D–S theory, for hypothesis A in FOD, the belief function Bel(A) and plausibility function Pl(A) are calculated according to the basic probability assignment BPA to form the belief interval [Bel(A),Pl(A)], which is used to indicate the degree of confirmation of hypothesis A.

### 2.2. Belief Entropy

Belief entropy is one of the hot issues in the field of information fusion, many types of belief entropy are proposed such as Dubois-Prade’s entropy [47], Jirousek-Shenoy entropy [48,49], Deng entropy [39], and so on [50]. Deng entropy as a measurement of uncertain information is defined as follows [39,51]:

(6) $$Ed\left(m\right)=-\sum_{A\in X}m\left(A\right)\log_{2}\frac{m\left(A\right)}{2^{\left|A\right|}-1},$$

among them, *m* is the a mass function defined on the FOD *X*, and *A* is a focal element of *m*. |A| stands for the cardinality of *A*.

### 2.3. Improved Base Belief Function

The improved base belief function is proposed to obtain the modified BPAs before data fusion in [45]. Let θ be a set of *N* possible values which are mutually exclusive. So, the power set of θ is $2^{\theta }$, where the number of elements is $2^{{}^{N}}$. If the FOD is complete, m(∅)=0. Determine the number of propositions with initial belief degree assigned in evidence group as λ. Thus, the improved base belief function $n(R_{i})$ is defined as [45]:

(7) $$n\left(R_{i}\right)=\frac{1}{\lambda }$$

where $R_{i}$ represents a subset in FOD Ω. λ represents the number of propositions with initial belief degree in evidence group. Then $n(R_{i})$ is adopted to modify the initial BPA *m* through the arithmetic mean [45]:

(8) $$m^{{}^{\prime }}\left(R_{i}\right)=\frac{n\left(R_{i}\right)+m\left(R_{i}\right)}{1+\frac{2^{\Omega }-1}{\lambda }}.$$

The following is an example of calculating the improved base belief function [45]. For FOD Ω={a,b,c}, the BPAs are as follows:

$$m_{1}\left(a\right)=0.99,m_{1}\left(a,b\right)=0.01;$$

$$m_{2}\left(b\right)=0.01,m_{2}\left(c\right)=0.99.\;\;\;\;\;$$

There are four focal elements {a}, {b}, {c} and {a,b} in $m_{1}$ and $m_{2}$. So, λ=4. Using Equation (7), the value of the improved base belief function is $n\left(R_{i}\right)=\frac{1}{\lambda }=\frac{1}{4}$. There are 3 elements in FOD, so the size is $2^{3}-1=7$. With Equation (8), the modified BPA are as follows:

$$\begin{array}{cccc} {m_{1}}^{{}^{\prime }}\left(a\right) & =\frac{m_{1}\left(a\right)+n\left(R_{i}\right)}{1+\frac{2^{\Omega }-1}{\lambda }}=\frac{0.99+\frac{1}{4}}{1+\frac{7}{4}}=0.45. & {m_{1}}^{{}^{\prime }}\left(a,b\right) & =\frac{m_{1}\left(a,b\right)+n\left(R_{i}\right)}{1+\frac{2^{\Omega }-1}{\lambda }}=\frac{0.01+\frac{1}{4}}{1+\frac{7}{4}}=0.09. \end{array}$$

$$\begin{array}{cc} {m_{1}}^{{}^{\prime }}\left(b\right) & =M_{1}\left(c\right)=M_{1}\left(a,c\right)=M_{1}\left(b,c\right)=M_{1}\left(a,b,c\right)=\frac{m_{1}\left(R_{i}\right)+n\left(R_{i}\right)}{1+\frac{2^{\Omega }-1}{\lambda }}=\frac{\frac{1}{4}}{1+\frac{7}{4}}=0.09. \end{array}$$

$$\begin{array}{cccc} {m_{2}}^{{}^{\prime }}\left(b\right) & =\frac{m_{2}\left(b\right)+n\left(R_{i}\right)}{1+\frac{2^{\Omega }-1}{\lambda }}=\frac{0.01+\frac{1}{4}}{1+\frac{7}{4}}=0.09. & {m_{2}}^{{}^{\prime }}\left(c\right) & =\frac{m_{2}\left(c\right)+n\left(R_{i}\right)}{1+\frac{2^{\Omega }-1}{\lambda }}=\frac{0.99+\frac{1}{4}}{1+\frac{7}{4}}=0.45. \end{array}$$

$$\begin{array}{cc} {m_{2}}^{{}^{\prime }}\left(a\right) & =M_{2}\left(a,b\right)=M_{2}\left(a,c\right)=M_{2}\left(b,c\right)=M_{2}\left(a,b,c\right)=\frac{m_{2}\left(c\right)+n\left(R_{i}\right)}{1+\frac{2^{\Omega }-1}{\lambda }}=\frac{0.99+\frac{1}{4}}{1+\frac{7}{4}}=0.09. \end{array}$$

## 3. Proposed Method

In this section, the improved base belief function in [45] and a belief entropy in [39] are adopted to construct a new data fusion method.

### 3.1. Method

**Figure 1:**
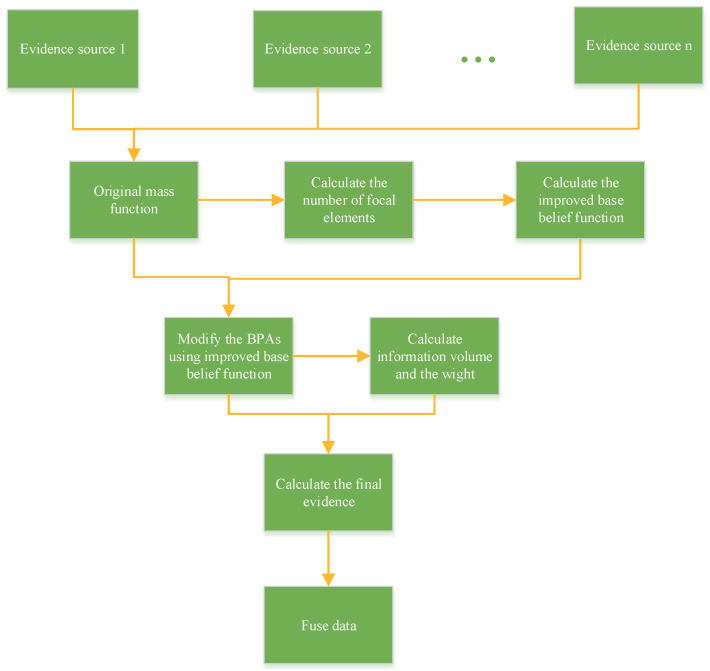
The flowchart of the proposed method.

The procedure of the proposed method is listed as follows. And the flowchart of the proposed improvement method is shown in Figure 1.

**Step 1:**  For potentially conflict data, the improved base belief function method *n* in Equation (7) is used to modify the BPAs to get the modified evidence $m^{\prime }$ of Equation (8).

Based on the improved base belief function, the situation where the belief of a proposition is zero can be avoided, which can overcome the shortcoming of Dempster combination rule in conflict data fusion.

**Step 2:**  For the *i*th evidence, the information volume Iv is calculated through the Deng entropy Ed(i) [39]. Iv is defined as follows:
  (9) $$\begin{array}{c} Iv\left(i\right)=e^{Ed}=e^{-\sum_{i}m\left(A_{i}\right)log\frac{m(A_{i})}{2^{|A_{i}|}-1}.} \end{array}$$

Calculating information volume is the basis of obtaining weight.

**Step 3:**  For each evidence, the weight w(i) is defined as follows:
  (10) $$\begin{array}{c} w\left(i\right)=\frac{Iv(i)}{\sum_{i=1}^{n}Iv\left(i\right)}. \end{array}$$

Due to different information sources have different influence on the final results, weight can represent the impact of each evidence group on the final result. In this way, each evidence group is assigned a small weight, which is more reasonable in application.

**Step 4:**  The weights are obtained through step 3 to modify the BPAs before fusing data. After evidence modification using the base belief function and information volume-based uncertainty, the final evidence for data fusion can be calculated as follows:
  (11) $$\begin{array}{c} m_{w}\left(A_{i}\right)=\sum_{i=1}^{n}w_{i}m_{i}^{\prime }\left(A_{i}\right). \end{array}$$

Using the weight factor to modify the BPAs again to get the final evidence. And fusing the final evidence can obtain better results which is more realistic.

**Step 5:**  The final evidence obtained by step 4 can be fused through the Dempster combination rule in Equation (4) to get the final result. If there are *n* bodies of evidence, then the modified evidence will be fused with n−1 times.
**Step 6:**  Decision making based on the data fusion result.

### 3.2. Examples and Discussion

Numerical examples are given to explain and verify the rationality of the proposed method.

**Example** **1.** *Supposed that the FOD is Ω={a,b} and the BPAs are given as*

$$\begin{array}{c} m_{1}\left(a\right)=1,m_{1}\left(b\right)=0,m_{1}\left(a,b\right)=0,\\ m_{2}\left(a\right)=0,m_{2}\left(b\right)=1,m_{2}\left(a,b\right)=0. \end{array}$$

*The improved base belief function based on Equation (7) is*

$$\begin{array}{c} n\left(a\right)=n\left(b\right)=n\left(a,b\right)=\frac{1}{3}. \end{array}$$

*Then, the improved base belief function is used to modify the BPAs based on Equation (8). The modified BPAs are*

$$\begin{array}{c} m_{1}\left(a\right)=0.6667,m_{1}\left(b\right)=0.1667,m_{1}\left(a,b\right)=0.1667,\\ m_{2}\left(a\right)=0.1667,m_{2}\left(b\right)=0.1667,m_{2}\left(a,b\right)=0.6667. \end{array}$$

*After evidence modification with the improved base belief function method, the information volume of E1 and E2 can be measured by belief entropy as:*

$$\begin{array}{c} Iv(E1)=2.8596,Iv(E2)=2.8596, \end{array}$$

*and the final evidence functions are*

$$\begin{array}{c} m(a)=0.4167,m(b)=0.1667,m(a,b)=0.3472. \end{array}$$

*Dempster combination rule is used for data fusion. The final result is:*

$$\begin{array}{c} m(a)=0.4787,m(b)=0.4787,m(a,b)=0.0426. \end{array}$$

In this example, the evidence sources given are completely conflict. According to the data of fusion result, the propositions {a} and {b} have an equal belief degree, which is consistent with the initial belief assignment in BPAs: $m_{1}\left(a\right)=m_{2}\left(b\right)=1$. In addition, a certain amount of belief is assigned to the proposition {a,b}, which shows the uncertainty among the propositions {a} and {b}.

**Example** **2.** *Supposed that the FOD is Ω={a,b,c} and two sets of BPAs (adopted from Zadeh [16,17]) are as follows.*

$$\begin{array}{c} m_{1}\left(a\right)=0.99,m_{1}\left(a,b\right)=0.01, \end{array}$$

$$\begin{array}{c} m_{2}\left(b\right)=0.01,m_{2}\left(c\right)=0.99. \end{array}$$

*The improved base belief function is*

$$\begin{array}{c} n\left(a\right)=n\left(b\right)=n\left(c\right)=n\left(a,b\right)=n\left(a,c\right)=n\left(b,c\right)=n\left(a,b,c\right)=\frac{1}{4} \end{array}$$

*Then, the modified BPAs using improved base belief function are:*

$$\begin{array}{c} m_{1}\left(a\right)=0.4509,m_{1}\left(a,b\right)=0.0945,m_{1}\left(b\right)=m_{1}\left(c\right)=m_{1}\left(a,c\right)=m_{1}\left(b,c\right)=m_{1}\left(a,b,c\right)=0.0909, \end{array}$$

$$\begin{array}{c} m_{2}\left(b\right)=0.0945,m_{2}\left(c\right)=0.4509,m_{2}\left(a\right)=m_{2}\left(a,b\right)=m_{2}\left(a,c\right)=m_{2}\left(b,c\right)=m_{2}\left(a,b,c\right)=0.0909. \end{array}$$

*After data modification, the information volume of E1 and E2 and the final evidence are:*

$$\begin{array}{c} Iv(E1)=7.9692,Iv(E2)=7.9375, \end{array}$$

$$\begin{array}{c} m(a)=0.2713,m(b)=0.0927, \end{array}$$

$$\begin{array}{c} m(c)=0.2706,m(a,b)=0.0927, \end{array}$$

$$\begin{array}{c} m(a,c)=m(b,c)=m(a,b,c)=0.0909. \end{array}$$

*The final evidence is calculated by Dempster combination rule. The final result is:*

$$\begin{array}{c} m(a)=0.3762,m(b)=0.1200, \end{array}$$

$$\begin{array}{c} m(c)=0.3729,m(a,b)=0.0400, \end{array}$$

$$\begin{array}{c} m(a,c)=m(b,c)=0.0389,m(a,b,c)=0.0129. \end{array}$$

According to the final result, a big belief degree for m(a) in $m_{1}$ and m(c) in $m_{2}$ is logical because {a} and {c} have a 99% belief assignment from the original evidence sources: $m_{1}\left(a\right)=m_{2}\left(c\right)=0.99$. And the proposition {b} does not get too much support of belief degree which is consistent with the initial belief assignment: $m_{1}\left(a,b\right)=0.01$ and $m_{2}\left(b\right)=0.01$. In addition, a certain amount of belief is assigned to the proposition {b,c}, {a,c} and {a,b,c}, which shows the uncertainty among the events {a}, {b} and {c}.

**Example** **3.** *Supposed that the FOD is Ω={a,b,c} and two sets of BPAs are given as*

$$\begin{array}{c} m_{1}\left(a\right)=0.9,m_{1}\left(a,b\right)=0.1, \end{array}$$

$$\begin{array}{c} m_{2}\left(c\right)=0.9,m_{2}\left(a,b,c\right)=0.1. \end{array}$$

*The improved base belief function is*

$$\begin{array}{c} n\left(a\right)=n\left(b\right)=n\left(c\right)=n\left(a,b\right)=n\left(a,c\right)=n\left(b,c\right)=n\left(a,b,c\right)=\frac{1}{4} \end{array}$$

*After evidence modification, the information volume of each evidence (E1 and E2) is:*

$$\begin{array}{c} Iv(E1)=8.6395,Iv(E2)=8.6395. \end{array}$$

**Figure 2:**
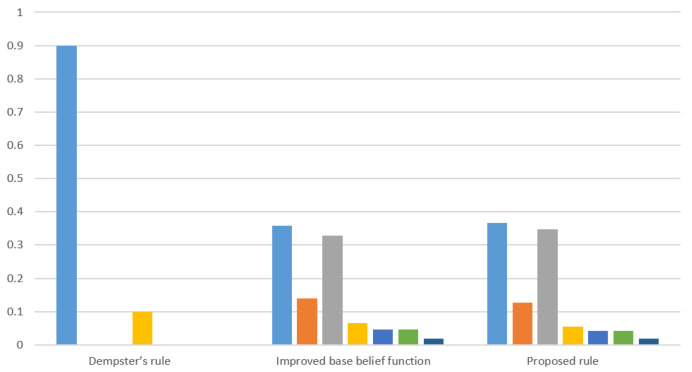
Comparison of fusion results using different methods in Example 3.

The final result can be calculated by Dempster combination rule, as shown in Table 1. The fusion result is compared with the methods with only classical Dempster combination rule and only the improved base belief function, as shown in Table 1 and Figure 2. From the original evidence, $m_{1}\left(a\right)=m_{2}\left(c\right)=0.9$, m(a) in $m_{1}$ and m(c) in $m_{2}$ have the same belief assignment for {a} and {c} as a single set, but, $m_{1}$ assigns no belief on {c}, thus, the proposed method assigns a higher belief on {a} than {c}. In addition, {b}, {a,c} and {b,c} which are assigned the belief degree, which shows the superiority of base belief assignment method in comparison with the method only using Dempster rule directly.

**Example** **4.** *Supposed that the FOD is Ω={a,b,c} and two sets of BPAs are given as*

$$\begin{array}{c} m_{1}\left(a\right)=0.9,m_{1}\left(b\right)=0.05,m_{1}\left(c\right)=0.05,\\ m_{2}\left(a\right)=0.05,m_{2}\left(b\right)=0.05,m_{2}\left(c\right)=0.9. \end{array}$$

**Figure 3:**
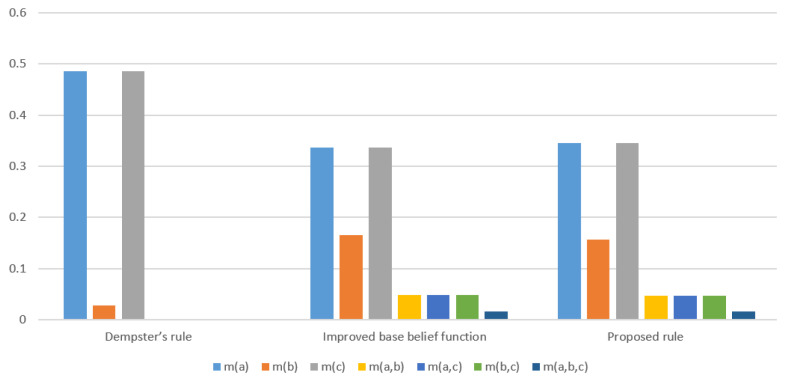
Comparison of fusion results using different methods in Example 4.

*The data fusion results with the proposed method and the methods with only classical Dempster combination rule and only improved base belief function are shown in Table 2 and Figure 3.*

Compared with the method only using Dempster combination rule, the proposed method can reflect the uncertainty among the events {a}, {b} and {c}. In addition, {a,b}, {a,c}, {b,c} and {a,b,c} also have belief assignment which are reasonable. While in comparison with the method only use the improved base belief function, the proposed method has more belief assignment on {a} and {c}. This is because in the initial BPAs, $m_{1}\left(a\right)=m_{2}\left(c\right)=0.9$, {a} and {c} have a significant higher belief than other propositions.

**Example** **5.** *Supposed that the FOD is Ω={a,b,c} and two sets of BPAs are given as*

$$\begin{array}{c} m_{1}\left(a\right)=0.9,m_{1}\left(a,b,c\right)=0.1, \end{array}$$

$$\begin{array}{c} m_{2}\left(a\right)=0.05,m_{2}\left(b\right)=0.05,m_{2}\left(c\right)=0.9. \end{array}$$

**Figure 4:**
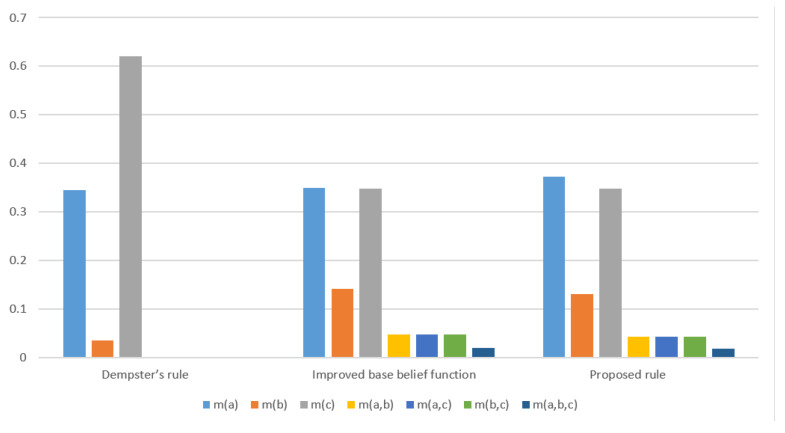
Comparison of fusion results using different methods in Example 5.

*The final results with the proposed method and the methods with only classical Dempster combination rule and improved base belief function are shown in Table 3 and Figure 4.*

Compared with the method only using Dempster combination rule, the proposed method can reflect the uncertainty among the events {a}, {b} and {c} reasonably. Compared with the method only using improved base belief function, the proposed method has more belief assignment on {a}. This is contributed by a belief assignment $m_{2}\left(a\right)=0.05$ on {a} in $m_{2}$ while no similar belief assignment on {c} in $m_{1}$. The uncertainty in multi subset proposition is reflected by assigning less belief degree on {c} in comparison with the method only use the improved base belief function, which also reflects the differences in the initial BPAs between this example and the previous one.

From the results of Examples 3, 4 and 5, the effectiveness and rationality of the proposed method for conflict data fusion are verified. Compared with the method with only Dempster combination rule or only improved base belief function, the proposed method can get a more rational fusion result. Because the proposed method considers both the initial belief assignment in base belief assignment and the information volume with belief entropy.

## 4. Application of Proposed Method

In this section, the classical example in machine learning to classify the Iris is adopted to evaluate the rationality and effectiveness of the proposed method. The real data set comes from the UCI machine learning library and the BPAs after evidence modelling are adopted from [44,52]. In the Iris data set, there are three species (named Setosa (a), Versicolor (b), and Virginica (c)), each species contains 50 instances. Each species of Iris has four attributes (sepal length (SL), sepal width (SW), petal length (PL), petal width (PW)).

### 4.1. Experiment 1

In [44], Wang et al. select 40 instances from each species randomly, so the remaining 10 are considered test sets. An instance is randomly selected from the species Setosa (a) of the test set to generate BPA. The BPAs of the four attributes are shown in Table 4.

**Figure 5:**
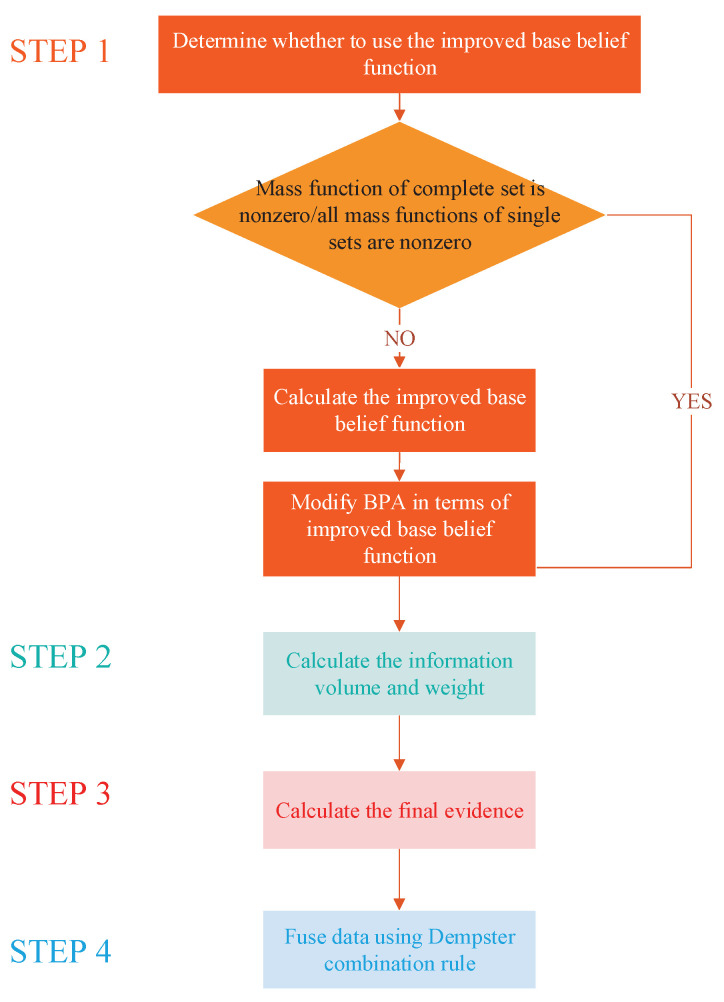
The calculation process of this experiment.

According to the steps of proposed method, the calculation procedure of this experiment is shown in Figure 5. The BPAs of the first two attributes (SL and SW) are assigned belief degree in all power set spaces, which does not lead to possible anti intuitive fusion results due to zero values when using Dempster composition rules. Therefore, only the BPAs of the last two attributes (PL and PW) will be modified using the proposed method. And the improved base belief function can be calculated as:

$$\begin{array}{c} n\left(a\right)=n\left(b\right)=n\left(c\right)=n\left(a,b\right)=n\left(a,c\right)=n\left(b,c\right)=n\left(a,b,c\right)=\frac{1}{7} \end{array}$$

According to the data modification steps based on the improved base belief function in the proposed method, the modification BPAs of the two attributes PL and PW is shown in Table 5.

After evidence modification, the information volume of each evidence is:

$$\begin{array}{c} Iv(E1)=4.1451,Iv(E2)=5.7692,Iv(3)=7.1579,Iv(E4)=7.2326 \end{array}$$

and the final evidence functions are

$$\begin{array}{c} m(a)=0.3770,m(b)=0.2449, \end{array}$$

$$\begin{array}{c} m(c)=0.1552,m(a,b)=0.0547, \end{array}$$

$$\begin{array}{c} m(a,c)=0.0546,m(b,c)=0.0632,m(a,b,c)=0.0504. \end{array}$$

After the BPAs of attribute PL and PW is modified based on the belief entropy, the final evidence is fused three times by using the Dempster combination rule, and the final result is calculated. Table 6 shows the final data fusion results using the proposed method and using only the improved base belief function. From the final results, the belief degree of the test case to Setosa (a) species is the highest, which is consistent with the actual situation, indicating the rationality of the proposed method. In addition, the belief degree using the proposed method assigned to species of Setosa (a) is 67.98%, which is higher than 62.32% using only the improved base belief function. According to this, the validity and rationality of the proposed method are shown.

### 4.2. Experiment 2

In [52], Yuan et al. took 120 specimens as the training set and the remaining 30 specimens as the test set to generate the BPAs. The generated BPAs of four attributes of Setosa samples are shown in Table 7, where θ means all the three species {a,b,c}.

All attributes of each sample have data conflicts, and only using Dempster composition rule will lead to possible illogical fusion results owing to zero values. So, according to the step 1 of the proposed method shown in Figure 5, all BPAs generated by four attributes of Setosa samples will be modified by using the improved base belief function firstly. Then the rest steps of the proposed method are executed to get the final evidence using belief entropy. The final combination results using the proposed method and only using the improved base belief function are shown in Table 8.

It can be seen from the combination results of two methods that the BPA of the proposition {*a*} is the highest in each sample. According to the final results, the sample is Setosa obviously. In addition, the BPA of hypothesis {*a*} using the proposed method is higher than only using improved base belief function. Compared with only using the improved base belief function, the proposed method can effectively deal with data conflicts to some extent. The results verify the validity and rationality of the proposed method.

## 5. Open Issues

Some open issues exist in the current work. First of all, the uncertainty measure in D–S theory is still an open issue. How to measure the reliable and independent of evidence needs further study. Is the belief entropy good enough for this open issue [33,39,50,51]?

Secondly, Dempster combination rule is axiomatically justified in [15,18,53]. Dempster rule can be used under the condition that two sources must be entirely reliable and independent. But this is also the source of problem, in practical world, there is full of uncertainty, it is hard to find two sources which are entirely reliable and independent. Among so many improved rules [29,54], how to find the proper one for the specific applications and cases?

The third is for information fusion in the open world assumption [30,31]. The unknown and new information should be taken into consideration. Dynamic evidence reasoning may be a choice [55].

The experiments should be conducted on several databases for further work.

## 6. Conclusions

Regarding conflict data fusion problems, an improved method is proposed in this paper, which is based on the belief entropy and improved base belief function in D–S theory. First, in the power set space of evidence, the initial belief is calculated through using the improved base belief function, and the initial belief is calculated according to the number of propositions with belief. Then, using the belief entropy measures the information volume of each evidence. The improved base belief function and information volume are used to modify the evidence. At last, the data fusion is based on Dempster combination rule. The effectiveness and rationality of the proposed method are verified by numerical examples and two applications of the proposed methed on classification data set.

The proposed method not only considers the focus elements which are assigned the initial belief in the current evidence, but also considers the proposition in the power set space such as the propositions which are zero value or are not assigned the belief degree. However, there are still some shortcomings. The proposed method only is applied to the closed-world hypothesis, however, the uncertain factors will increase in the open-world.

## Figures and Tables

**Table 1:** Results of three combination methods of Example 3.

Method	*m*(*a*)	*m*(*b*)	*m*(*c*)	*m*(a,b)	*m*(a,c)	*m*(b,c)	*m*(a,b,c)
Dempster’s rule	0.9	0	0	0.1	0	0	0
Improved base belief function [45]	0.3587	0.1405	0.3278	0.0656	0.0468	0.0468	0.0193
Proposed method	0.3669	0.1278	0.3479	0.0541	0.0426	0.0426	0.0180

**Table 2:** Results of three combination methods of Example 4.

Method	*m*(*a*)	*m*(*b*)	*m*(*c*)	*m*(a,b)	*m*(a,c)	*m*(b,c)	*m*(a,b,c)
Dempster’s rule	0.4865	0.0270	0.4865	0	0	0	0
Improved base belief function [45]	0.3365	0.1653	0.3365	0.0485	0.0485	0.0485	0.0162
Proposed method	0.3446	0.1571	0.3446	0.0461	0.0461	0.0461	0.0154

**Table 3:** Results of three combination methods of Example 5.

Method	*m*(*a*)	*m*(*b*)	*m*(*c*)	*m*(a,b)	*m*(a,c)	*m*(b,c)	*m*(a,b,c)
Dempster’s rule	0.3448	0.0345	0.6207	0	0	0	0
Improved base belief function [45]	0.3502	0.1418	0.3480	0.0469	0.0469	0.0469	0.0193
Proposed method	0.3734	0.1302	0.3481	0.0433	0.0433	0.0433	0.0184

**Table 4:** BPAs of four attributes.

Attribute	*m*(*a*)	*m*(*b*)	*m*(*c*)	*m*(a,b)	*m*(a,c)	*m*(b,c)	*m*(a,b,c)
SL	0.3337	0.3165	0.2816	0.0307	0.0052	0.0272	0.0052
SW	0.3164	0.2501	0.2732	0.0304	0.0481	0.0515	0.0304
PL	0.6699	0.3258	0	0	0	0.0043	0
PW	0.6996	0.2778	0	0	0	0.0226	0

**Table 5:** Modified BPAS of four attributes.

Attribute	*m*(*a*)	*m*(*b*)	*m*(*c*)	*m*(a,b)	*m*(a,c)	*m*(b,c)	*m*(a,b,c)
SL	0.3337	0.3165	0.2816	0.0307	0.0052	0.0272	0.0052
SW	0.3164	0.2501	0.2732	0.0304	0.0481	0.0515	0.0304
PL	0.4064	0.2343	0.0714	0.0714	0.0714	0.0736	0.0714
PW	0.4212	0.2103	0.0714	0.0714	0.0714	0.0827	0.0714

**Table 6:** Experiment results of different combination rules in Iris recognition.

Method	*m*(*a*)	*m*(*b*)	*m*(*c*)	*m*(a,b,c)
Improved base belief function [45]	0.6232	0.2671	0.1083	0
Proposed method	0.6798	0.2385	0.0869	0

**Table 7:** BPAs of four attributes of Setosa samples.

	PL	PW	SL	SW
Sample 1	m(a) = 0.6486	m(a) = 0.7477	m(a) = 0.8650	m(a) = 0
	m(b) = 0	m(b) = 0	m(b) = 0	m(b) = 0.9000
	m(a,b) = 0	m(a,b) = 0	m(a,b) = 0.0821	m(a,b) = 0
	m(b,c) = 0	m(b,c) = 0	m(b,c) = 0	m(b,c) = 0.1
	m(θ) = 0.3514	m(θ) = 0.2523	m(θ) = 0.0529	m(θ) = 0
Sample 2	m(a) = 0.6486	m(a) = 0.7477	m(a) = 0.2712	m(a) = 0
	m(b) = 0	m(b) = 0	m(b) = 0	m(b) = 0.9000
	m(a,b) = 0	m(a,b) = 0	m(a,b) = 0	m(a,b) = 0
	m(b,c) = 0	m(b,c) = 0	m(b,c) = 0	m(b,c) = 0.1
	m(θ) = 0.3514	m(θ) = 0.2523	m(θ) = 0.7288	m(θ) = 0
Sample 3	m(a) = 0.6486	m(a) = 0.7547	m(a) = 0.1356	m(a) = 0
	m(b) = 0	m(b) = 0	m(b) = 0	m(b) = 0.9000
	m(a,b) = 0	m(a,b) = 0	m(a,b) = 0	m(a,b) = 0
	m(b,c) = 0	m(b,c) = 0	m(b,c) = 0	m(b,c) = 0.1
	m(θ) = 0.3514	m(θ) = 0.2453	m(θ) = 0.8644	m(θ) = 0
Sample 4	m(a) = 0.6857	m(a) = 0	m(a) = 0.8650	m(a) = 0
	m(b) = 0	m(b) = 0	m(b) = 0	m(b) = 0.9000
	m(a,b) = 0	m(a,b) = 0	m(a,b) = 0.0821	m(a,b) = 0
	m(b,c) = 0	m(b,c) = 0	m(b,c) = 0	m(b,c) = 0.1
	m(θ) = 0.3143	m(θ) = 1	m(θ) = 0.0529	m(θ) = 0
Sample 5	m(a) = 0	m(a) = 0.3738	m(a) = 0.7560	m(a) = 0
	m(b) = 0	m(b) = 0	m(b) = 0	m(b) = 0.9000
	m(a,b) = 0	m(a,b) = 0	m(a,b) = 0.1484	m(a,b) = 0
	m(b,c) = 0	m(b,c) = 0	m(b,c) = 0	m(b,c) = 0.1
	m(θ) = 1	m(θ) = 0.6262	m(θ) = 0.0956	m(θ) = 0
Sample 6	m(a) = 0.8649	m(a) = 0.7477	m(a) = 0.6780	m(a) = 0
	m(b) = 0	m(b) = 0	m(b) = 0	m(b) = 0.9000
	m(a,b) = 0	m(a,b) = 0	m(a,b) = 0	m(a,b) = 0
	m(b,c) = 0	m(b,c) = 0	m(b,c) = 0	m(b,c) = 0.1
	m(θ) = 0.1351	m(θ) = 0.2523	m(θ) = 0.3220	m(θ) = 0
Sample 7	m(a) = 0.6857	m(a) = 0.7547	m(a) = 0.7560	m(a) = 0
	m(b) = 0	m(b) = 0	m(b) = 0	m(b) = 0.9000
	m(a,b) = 0	m(a,b) = 0	m(a,b) = 0.1484	m(a,b) = 0
	m(b,c) = 0	m(b,c) = 0	m(b,c) = 0	m(b,c) = 0.1
	m(θ) = 0.3143	m(θ) = 0.2453	m(θ) = 0.0956	m(θ) = 0
Sample 8	m(a) = 0.8649	m(a) = 0.7547	m(a) = 0.4068	m(a) = 0
	m(b) = 0	m(b) = 0	m(b) = 0	m(b) = 0.9000
	m(a,b) = 0	m(a,b) = 0	m(a,b) = 0	m(a,b) = 0
	m(b,c) = 0	m(b,c) = 0	m(b,c) = 0	m(b,c) = 0.1
	m(θ) = 0.1351	m(θ) = 0.2453	m(θ) = 0.5932	m(θ) = 0
Sample 9	m(a) = 0.9143	m(a) = 0.7547	m(a) = 0.5253	m(a) = 0
	m(b) = 0	m(b) = 0	m(b) = 0	m(b) = 0.9000
	m(a,b) = 0	m(a,b) = 0	m(a,b) = 0.2887	m(a,b) = 0
	m(b,c) = 0	m(b,c) = 0	m(b,c) = 0	m(b,c) = 0.1
	m(θ) = 0.0857	m(θ) = 0.2453	m(θ) = 00.1860	m(θ) = 0
Sample 10	m(a) = 0.8649	m(a) = 0.7547	m(a) = 0.8650	m(a) = 0
	m(b) = 0	m(b) = 0	m(b) = 0	m(b) = 0.9000
	m(a,b) = 0	m(a,b) = 0	m(a,b) = 0.0821	m(a,b) = 0
	m(b,c) = 0	m(b,c) = 0	m(b,c) = 0	m(b,c) = 0.1
	m(θ) = 0.1351	m(θ) = 0.2453	m(θ) = 0.0529	m(θ) = 0

**Table 8:** The final results of two different method.

		m(a)	m(b)	m(c)	m(a,b)	m(a,c)	m(b,c)	m(θ)
Sample 1	Improved base belief function	0.6158	0.2641	0.1014	0.0258	0.1014	0.0134	0.0017
	Proposed method	0.6639	0.2259	0.0862	0.0300	0.0099	0.0120	0.0022
Sample 2	Improved base belief function	0.4781	0.3376	0.1346	0.0299	0.0193	0.0255	0.0051
	Proposed method	0.5303	0.2690	0.1229	0.0394	0.0218	0.0253	0.0092
Sample 3	Improved base belief function	0.4604	0.3549	0.1401	0.0310	0.0207	0.0273	0.0056
	Proposed method	0.4992	0.2853	0.1316	0.0422	0.0251	0.0290	0.0118
Sample 4	Improved base belief function	0.4811	0.3378	0.1295	0.0281	0.0167	0.0217	0.0035
	Proposed method	0.4893	0.2999	0.1283	0.0459	0.0240	0.0279	0.0110
Sample 5	Improved base belief function	0.3875	0.4038	0.1404	0.0329	0.0214	0.0281	0.0055
	Proposed method	0.4216	0.3237	0.1410	0.0557	0.0301	0.0345	0.0168
Sample 6	Improved base belief function	0.6168	0.2589	0.1070	0.0260	0.0116	0.0151	0.0022
	Proposed method	0.6639	0.2209	0.0897	0.0278	0.0107	0.0129	0.0025
Sample 7	Improved base belief function	0.6057	0.2868	0.1001	0.0278	0.0104	0.0135	0.0018
	Proposed method	0.6623	0.2305	0.0836	0.0326	0.0096	0.0115	0.0021
Sample 8	Improved base belief function	0.5658	0.2973	0.1192	0.0281	0.0144	0.0187	0.0030
	Proposed method	0.6021	0.2469	0.1061	0.0336	0.0157	0.0185	0.0050
Sample 9	Improved base belief function	0.6304	0.3157	0.0913	0.0315	0.0087	0.0112	0.0013
	Proposed method	0.6614	0.2443	0.0744	0.0377	0.0077	0.0093	0.0014
Sample 10	Improved base belief function	0.6680	0.2355	0.0902	0.0251	0.0080	0.0103	0.0011
	Proposed method	0.7023	0.2098	0.0748	0.0254	0.0071	0.0087	0.0011
